# Clinical features of Q fever confirmed by plasma metagenomic next-generation sequencing

**DOI:** 10.3389/fimmu.2026.1847365

**Published:** 2026-07-08

**Authors:** Man Chen, Huaying Zhou, Zhuyun Zhou, Yan He, Yongfang Jiang

**Affiliations:** 1 Department of Infectious Diseases, The Second Xiangya Hospital, Central South University, Changsha, Hunan, China; 2 FuRong Laboratory, Central South University, Changsha, Hunan, China; 3 Clinical Medical Research Center for Viral Hepatitis in Hunan Province, Changsha, Hunan, China

**Keywords:** antiphospholipid antibodies, *Coxiella burnetii*, metagenomic next-generation sequencing, prolonged fever, Q fever

## Abstract

**Background:**

The clinical features of acute Q fever and their link to transient autoantibodies remain poorly defined. We characterized 44 plasma mNGS-confirmed cases and identified predictors of prolonged fever.

**Methods:**

Retrospective study (2021–2026) of 44 patients with confirmed Q fever (mNGS + clinical + exposure criteria). Patients were grouped by post-treatment fever duration (>7 days vs. ≤7 days).

**Results:**

Cohort was predominantly middle-aged (median 52.5 years) and male (95.5%). Common presentations: fever (95.5%), headache (45.5%), pulmonary involvement (51.2%). Elevated CRP (97.7%) and ESR (90.9%) were universal. Transient antiphospholipid antibodies (78.9%, all negative at 12 weeks) and ANA (23.5%) were frequent. Prolonged fever (>7 days) was associated with higher WBC, CRP, ESR, lower CD8+ and B cells, and aPL positivity (all p<0.05).

**Conclusions:**

Acute Q fever frequently induces transient autoantibodies. Prolonged fever correlates with an inflammatory and lymphopenic phenotype, underscoring immune dysregulation in recovery.

## Introduction

Q fever is a zoonotic disease caused by Coxiella burnetii, an obligate intracellular bacterium with a worldwide distribution (1). Domestic ruminants such as cattle, sheep, and goats serve as the primary reservoirs, and human infection typically occurs through inhalation of aerosolized bacteria from contaminated environments or animal products (2). The clinical presentation of Q fever is highly variable, ranging from asymptomatic seroconversion to self-limited febrile illness, pneumonia, hepatitis, and, in chronic cases, life-threatening endocarditis or vascular infections (3, 4). This protean nature often leads to diagnostic delays and under-recognition, particularly in non-endemic regions.

Traditionally, the diagnosis of Q fever has relied on serological testing (detecting antibodies against phase I and phase II antigens) and polymerase chain reaction (PCR) for C. burnetii DNA (5). However, these methods have limitations: seroconversion may take 2–3 weeks, and PCR sensitivity varies depending on sample type and disease stage (6). In recent years, metagenomic next-generation sequencing (mNGS) has emerged as a powerful diagnostic tool for infectious diseases, offering unbiased pathogen detection directly from clinical specimens (7). Plasma mNGS is particularly valuable for detecting fastidious or intracellular organisms such as C. burnetii, especially when conventional tests are negative or unavailable (8).

Although the clinical features of acute Q fever have been described in various cohorts, most studies have relied on serological diagnosis and may not fully capture the disease spectrum in the era of molecular diagnostics (9–11). Moreover, while most patients with acute Q fever respond well to doxycycline therapy, the duration of fever after treatment initiation is highly variable. A subset of patients experience prolonged fever lasting beyond one week despite appropriate anti-rickettsial therapy (12), yet the factors predicting prolonged fever remain poorly characterized. Understanding these risk factors could inform clinical management and identify patients who may benefit from closer monitoring or adjunctive therapies.

Emerging evidence suggests that host immune responses play a critical role in determining the clinical course of Q fever (13). C. burnetii modulates host immunity through various mechanisms, including evasion of phagolysosomal fusion and manipulation of cytokine responses (14). Interestingly, acute Q fever has been associated with transient autoimmunity phenomena, such as the production of antiphospholipid antibodies and antinuclear antibodies, which may complicate diagnosis and mimic autoimmune diseases (15, 16). However, the relationship between these autoimmunity markers and clinical outcomes, including fever resolution, has not been systematically investigated.

In this study, we employed plasma mNGS for definitive diagnosis to comprehensively characterize the clinical, laboratory, and microbiological features of Q fever in a cohort of 44 patients. We specifically aimed to identify risk factors associated with prolonged fever after initiation of targeted antibiotic therapy, with a particular focus on inflammatory markers, lymphocyte subsets, and autoimmunity markers. Elucidating these associations may provide insights into the pathophysiology of delayed recovery and guide personalized management strategies for patients with acute Q fever.

## Patients and methods

### Study design and population

This retrospective study analyzed the medical records of patients with Q fever confirmed by plasma mNGS at the Second Xiangya Hospital, Central South University (Changsha, China) between March 2021 and January 2026. Patients were eligible for inclusion if they met the following criteria: (1) underwent plasma mNGS testing, and (2) were suspected of having Q fever. Patients were excluded if they had (1) incomplete medical records or (2) no available mNGS results. For all included patients, plasma mNGS and conventional microbiological tests (CMTs) were performed concurrently. Detailed laboratory procedures are provided in the dedicated section (Plasma mNGS and CMT Procedures). At our center, plasma mNGS was primarily ordered for patients with suspected infections of unclear etiology after negative or inconclusive conventional microbiological tests, those with severe or atypical presentations, or those who failed empirical therapy.

### Diagnostic criteria for mNGS-confirmed Q fever

A patient was classified as Q fever only if all of the following were present: (i) detection of Coxiella burnetii reads by plasma mNGS; (ii) clinically compatible manifestations (fever, hepatitis, pneumonia, or nonspecific flu-like symptoms); and (iii) a plausible epidemiologic exposure history (contact with livestock, unpasteurized dairy products, or agricultural environments) within the incubation period. In the absence of a clear exposure history, confirmation required consistent clinical features and the exclusion of alternative infections by conventional microbiological tests (CMTs). No independent serological or PCR confirmation was available for this retrospective cohort, and mNGS positivity alone was not considered sufficient for diagnosis. Thus, final case assignment relied on integrated clinical, epidemiologic, and microbiologic evidence.

This study was approved by the Research Ethics Committee of the Second Xiangya Hospital, and the requirement for written informed consent was waived due to its retrospective design.

### Clinical data collection

Data were retrieved from patients’ electronic medical records. The following variables were collected at the time of diagnosis: Demographics, presenting symptoms, physical findings, laboratory results (obtained within 48 hours before initiation of anti-infective therapy), imaging findings, anti-infective treatment regimens, and outcomes. Primary outcome was fever duration after initiation of specific anti-infective therapy. Fever duration was calculated as the number of days from the start of the first dose of doxycycline (or alternative anti-rickettsial therapy if doxycycline was contraindicated) to the day of defervescence. Prolonged fever was defined *a priori* as fever duration >7 days after treatment initiation, based on the typical clinical response of acute Q fever to doxycycline (expected defervescence within 3–7 days). Secondary outcomes included in-hospital mortality, development of complications (hepatitis, pneumonitis, meningoencephalitis), and positivity for autoimmunity markers. Because serological testing for C. burnetii phase I/II antibodies and specific polymerase chain reaction (PCR) were not part of routine clinical practice at our center during the study period, these data were not systematically available and were therefore not used for inclusion.

### Sample processing and DNA extraction for mNGS

Specimens were collected from patients using standard procedures. Briefly, 3 mL of blood was drawn into sterile, DNase-free tubes. Plasma was separated within 8 hours of collection by centrifugation at 1,600×g for 10 minutes at 4 °C. Then, 300 μL of the plasma was transferred to a 2-mL centrifuge tube for nucleic acid extraction. Nucleic acids were extracted using a magnetic bead-based DNA/RNA extraction kit (Genskey Medical Technology Co., Ltd., Beijing, China) according to the manufacturer’s standard protocol.

### Library preparation and sequencing

The DNA library was constructed using a standard Illumina- compatible protocol, including DNA fragmentation, end repair, adapter ligation, and PCR amplification. Library quality was assessed on an Agilent 2100 Bioanalyzer. The prepared library was then denatured and diluted according to the manufacturer’s instructions. Finally, sequencing was performed on the Illumina NextSeq 550Dx platform (Illumina, Inc.).

### Bioinformatic analysis

Low-quality reads and those shorter than 35 bp were filtered out using fastp (17), generating high-quality sequencing data (≥10 million reads). Human host sequences were then removed by aligning the reads to the human reference genome (hg19) with the Burrows-Wheeler Aligner (BWA) (18). After discarding low-complexity reads, the remaining data were aligned against an in-house Pathogen Metagenomics Database (PMDB) covering bacteria, fungi, viruses, and parasites. The reference sequences for taxonomic classification were retrieved from NCBI RefSeq and included 11,958 bacterial genomes or scaffolds, 1,714 fungi associated with human infection, 7,373 complete viral genome sequences, and 343 parasites pathogenic to humans. Coverage ratios and depths for each microorganism were calculated using BEDTools (19).

The following in-house criteria were applied to define a positive mNGS finding for a pathogen. For bacteria, viruses, and parasites, a microbe at the species level was considered positive if its coverage rate was at least ten times that of any other microbe in the same category. For fungi, owing to their typically lower biomass in DNA extraction, a species-level fungus was considered positive when its coverage rate was five times higher than that of any other fungus. For Coxiella burnetii, given the inherent difficulty of DNA extraction and the low likelihood of exogenous contamination, the presence of a single read mapped to either the species or genus level was accepted as positive.

### Statistical Analysis

Normality was assessed using the Shapiro-Wilk test; non-normally distributed continuous variables are presented as median (IQR) and compared using the Mann-Whitney U test. Categorical variables are expressed as frequencies and percentages, compared by chi-square test (or Fisher’s exact test when expected frequencies <5). Given the exploratory nature and small sample size, no correction for multiple comparisons was applied, and p-values are descriptive only. Multivariable analysis was not performed due to insufficient sample size. All analyses used IBM SPSS Statistics 26.0.

## Results

### Patient profiles

**Table 1** summarizes the demographic characteristics of the 44 enrolled patients with Q fever. Acute infections accounted for 41 cases (93.2%). The median age was 52.5 years (range: 19–68 years), and 63.6% of the patients were over 50 years old. The cohort was predominantly male (42 patients, 95.5%). Diabetes and hypertension were observed in 5 (11.3%) and 8 (18.2%) patients, respectively, while chronic liver disease was present in 4 patients (9.1%). Exposure history included agricultural/residential exposure in 18 (40.9%), livestock contact in 14 (31.8%), and no clear exposure in 12 (27.3%).The most common presenting symptoms included fever, muscle and joint pain, and headache, reported in 42 (95.5%), 14 (31.8%), and 20 patients (45.5%), respectively. Cough was noted in 10 patients (22.7%). Hepatosplenomegaly and splenomegaly were observed in 10 of 41 (24.4%) and 18 of 41 (43.9%) patients, respectively. The lungs were the most common site of involvement, observed in 21 patients (51.2%, 21/41), with segmental or lobar opacities affecting the upper or lower lobes being the most frequent findings.

**Table 1:** Demographic characteristics.

Clinical characteristics n (%)	All patients (n = 44)
Age, years	52.5 (38.25, 57.0)
Age >50 years	28 (63.6)
Male	42 (95.5%)
Comorbidities	
Diabetes	5 (11.3%)
Hypertension	8 (18.2%)
History of tumor	4 (9.1%)
Chronic liver disease	4 (9.1%)
Nephrolithiasis	3 (6.8%)
Past surgical history	6 (13.6%)
Exposure history, n/N (%)	
Agricultural/residential exposure (farming, rural area)	18 (40.9%)
Contact with livestock	14 (31.8%)
No clear exposure identified	12 (27.3%)
Symptoms	
Fever	42 (95.5%)
Cough	10 (22.7%)
Muscle and joint pain	14 (31.8%)
Headache	20 (45.5%)
Hepatosplenomegaly	10/41
Splenomegaly	18/41
Peritoneal effusion	17 (38.6%)
Pleural effusion	11 (25%)
Pneumonia (Segmental or lobar, lobe involvements)	21/41
Acute Q fever	41 (93.2%)

Peritoneal effusion and pleural effusion were present in 17 (38.6%) and 11 (25.0%) patients, respectively. Three patients in our study were diagnosed with chronic Q fever: one with chronic endocarditis, one with a vascular infection, and one with Q fever peritonitis. The third patient had chronic renal failure and was on long-term peritoneal dialysis.

**Table 2** outlines the most frequently observed laboratory findings in the study population. The most common laboratory abnormalities were elevated C-reactive protein (CRP) and erythrocyte sedimentation rate (ESR), present in 43 (97.7%) and 40 (90.9%) patients, respectively. Procalcitonin (PCT) levels >1 ng/mL were observed in 14 patients (31.8%). Most patients had normal leukocyte counts; leukocytosis was observed in 10 patients (22.7%), and leukopenia in 2 patients (4.5%). Atypical lymphocytes (Downey cells) were found in 9 patients (20.5%). Anemia and thrombocytopenia were each present in 5 patients (11.3%). Most patients had elevated liver enzymes, with total bilirubin (TB) >5 times the upper limit of normal (ULN) in 3 patients (6.8%). Elevated creatinine was observed in 11 patients (25.0%). A 5-fold increase in D-dimer levels (above ULN) was found in 23 of 34 patients (67.6%). Assessment of autoimmunity markers during the acute phase revealed positive antiphospholipid antibodies in 15 of 19 patients tested (78.9%); among these 15 patients, none had thrombotic events, 12 retested at 12 weeks became negative. In addition, positive antinuclear antibodies (ANA) were detected in 8 of 34 patients tested (23.5%). Plasma mNGS was performed in all patients, and the specific reads of Coxiella burnetii ranged from 1 to 3,184. Peritoneal dialysis fluid mNGS was performed in one patient, in whom the number of Coxiella burnetii reads was 452,513.

**Table 2:** Laboratory findings, treatment, and mNGS timing in 44 patients with Q fever.

Clinical characteristics [range, n (%)]	All patients (n = 44)
Leukocytosis	10 (22.72%)
Leukopenia	2 (4.5%)
Downey cells	9 (20.5%)
Anemia	5 (11.3%)
Thrombocytopenia	5 (11.3%)
Increased CRP	43 (97.7%)
Increased ESR	40 (90.9%)
PCT>1 ng/ml	14 (31.8%)
Elevated ALT	39 (88.6%)
Elevated AST	31 (70.5%)
Elevated TB	9 (20.5%)
TB>5ULN	3 (6.8%)
Elevated creatinine	11 (25%)
D-Dimer >5 ULN	23/34
Antiphospholipid taken	19
Positive Antiphospholipid results	15 (78.9%)
Positive antinuclear antibodies	8/34
mNGS reads	1-3184
Duration of fever after targeted antibiotic (d)	1-33
Doxycycline initiated, n/N (%)	44/44 (100)
Time from symptom onset to doxycycline, median (range), days	10 (5–62)
Alternative antibiotics (before diagnosis), n/N (%)	14/44 (27.3)
Days from admission to mNGS result, median (range)	2 (1–9)

CRP, C-reactive protein; ESR, erythrocyte sedimentation rate; PCT, Procalcitonin; ALT, alanine aminotransferase; AST, Aspartate aminotransferase; TB, total bilirubin; ULN, upper limit of normal; mNGS, metagenomic next- generation.

Five patients with severe headache underwent lumbar puncture; their cerebrospinal fluid (CSF) findings are presented in **Table 3**. Abnormal findings included modestly elevated CSF opening pressure in three patients, mildly elevated CSF protein in one patient, and minimally increased CSF white blood cell (WBC) count in one patient. CSF glucose and chloride levels were within normal limits in all patients.

**Table 3:** Cerebrospinal fluid characteristics of five patients with Q fever.

Normal range	Color	Proteins, mg/L	Glucose, mmol/L	Chloride, mmol/L	Pressure, mm H2O	WBC count, cells/µL	WBC types
Patient 1	Clear	370	4.09	123.6	120	2	Lymphocytes
Patient 2	Clear	540	4.41	119	225	0	
Patient 3	Clear	351	3.65	120.9	265	0	
Patient 4	Clear	360	3.66	122.5	120	25	Lymphocytes
Patient 5	Clear	214	3.49	117.3	220	0	
Normal range	Clear	150-450	2.2 – 3.9	118 – 132	80–180	0 – 5	

### Risk factors for extended fever resolution following targeted antibiotic therapy

All 44 patients (100%) received doxycycline, with a median time from symptom onset to doxycycline initiation of 10 days (range: 5–62 days). Before diagnosis, 14 patients (27.3%) had received alternative antibiotics. The median duration of fever after initiation of targeted antibiotics (doxycycline or alternatives) was 8 days (range: 1–33 days). Patients were stratified into two groups according to fever resolution time: prolonged fever (>7 days) and non-prolonged fever (≤7 days). Regarding mNGS testing, the median time from hospital admission to the mNGS result was 2 days (range: 1–9 days) (**Table 3**).

As shown in **Table 4**, patients in the prolonged fever group (n =22) compared to the non-prolonged group (n = 19) had significantly higher white blood cell counts (median [IQR]: 9.04 [7.25-11.37] vs. 6.03 [4.48-8.2] ×10^9^/L, p = 0.004), C-reactive protein (CRP: 74.35 [52.11-117.52] vs. 47.53 [31.09-81.74] mg/L, p = 0.045), and erythrocyte sedimentation rate (ESR: 44 [22.5-64] vs. 33 [17.5-42.25] mm/h, p = 0.042). Conversely, they had lower CD8+ T lymphocyte counts (398 [206.75-643] vs. 582.5 (245–2204) cells/μL, p=0.040) and B lymphocyte counts (151 [111.5- 158.75] vs. 221 [207-298.5] cells/μL, p = 0.049). The proportion of patients positive for antiphospholipid antibodies was also higher in the prolonged fever group (10/10 [100%] vs. 4/7 [57%] (p = 0.023).

**Table 4:** Risk factors for extended fever resolution following targeted antibiotic therapy.

Characteristics [median (IQR) or n (%)]	Prolonged (>7 d) n=22	Shortened (≤7 d) n=19	P
Age (years)	51.5 (40.75-55.25)	53 (36-58)	0.856
Male, n (%)	21 (95.5%)	18 (94.7%)	0.918
WBC (109/L)	9.04 (7.25-11.37)	6.03 (4.48-8.2)	**0.004**
PLT (109/L)	203.5 (118.75-306.25)	159 (130-240)	0.344
CRP (mg/l)	74.35(52.11-117.52)	47.53 (31.09-81.74)	0.045
PCT (ng/ml)	0.8 (0.54-1.32)	0.69 (0.42-0.98)	0.717
ALT (U/L)	95.35 (69.45-127.03)	99.3 (52.6-112.1)	0.598
AST (U/L)	78.2 (38.25-100.95)	60.6 (43.8-87.4)	0.384
TB (µmol/L)	12.75 (11.15-24.8)	10.9 (9.2-15)	0.563
Cr (µmol/L)	71.5 (59.68-82.75)	83 (72.7-94.5)	0.152
ESR	44 (22.5-64)	33 (17.5-42.25)	0.042
D-Dimer	3.54 (2.26-6.13)	2.69 (1.79-6.36)	0.553
mNGS reads	9 (2-21.5)	11 (2-84)	0.752
CD4 T cell (106/L)	727.5 (384.25-829.25)	800 (671.5-1068.25)	0.402
CD8 T cell (10^6^/L)	398 (206.75-643)	582.5 (245-2204))	**0.040**
NK cell (10^6^/L)	144 (85.75-246.5)	253.5 (217.75-291.25)	0.107
B lymphocyte (10^6^/L)	151(111.5-158.75)	221 (207-298.5)	**0.049**
CD4/CD8	1.23 (0.99-3.79)	1.04 (0.47-2.70)	0.565
aPL positivity	10/10 (100%)	4/7 (57%)	**0.023**

Data are presented as No. (%) or median (interquartile range) unless otherwise indicated. Values in bold indicate statistical significance.

IQR, interquartile range; WBC, White Blood Cell Count; PLT, Platelet; CRP, C-reactive protein; PCT, Procalcitonin; ALT, alanine aminotransferase; AST, Aspartate aminotransferase; TB, total bilirubin; Cr, Creatinine; ESR, erythrocyte sedimentation rate; mNGS, metagenomic next- generation; NK, Natural Killer; aPL, Antiphospholipid antibodies.

Although CD4+ T cell and NK cell counts were lower in the prolonged group, the differences did not reach statistical significance (p =0.402 and p = 0.107, respectively). No significant differences were observed between the two groups in age, male sex, aspartate aminotransferase (AST), alanine aminotransferase (ALT), total bilirubin (TB), hemoglobin, platelet count, creatinine, D-dimer levels, or C. burnetii-specific mNGS read counts (p > 0.05 for all; see **Table 4** for full details).

## Discussion

In this study, we comprehensively analyzed 44 patients with Q fever, focusing on risk factors for prolonged fever after targeted antibiotic therapy. To our knowledge, this is one of the few studies evaluating the relationship between host immune responses, autoimmunity markers, and delayed fever resolution in acute Q fever.

Demographics and clinical manifestations. Our cohort was predominantly middle-aged males, consistent with previous reports (1, 3). Clinical manifestations ranged from self-limited febrile illness to pulmonary involvement and hepatitis, underscoring the protean nature of acute Q fever (4).

Inflammatory and laboratory markers. Nearly all patients had elevated CRP (97.7%) and ESR (90.9%). These acute-phase reactants were associated with fever and systemic symptoms. This pattern reflects the potent immunostimulatory properties of Coxiella burnetii. The bacterium triggers innate immune responses primarily through recognition of bacterial lipoproteins by the TLR1/TLR2 heterodimer and the cytosolic receptor NOD2, leading to MyD88-dependent production of pro-inflammatory cytokines (TNF-α, IL-6) and immunomodulatory cytokines (IL-10, TGF-β) (14, 23). Leukocytosis was present in only 22.7%, and PCT >1 ng/mL in 31.8% (median 0.81 ng/mL). This pattern-intense inflammation without marked leukocytosis-may help differentiate Q fever from common bacterial infections. Atypical lymphocytes (Downey cells) were noted in 20.5%, a finding that could cause diagnostic confusion with viral illnesses.

Transient autoantibodies. aPL was positive in 15/19 (78.9%). None had thrombotic events, and all 12 retested at 12 weeks became negative, confirming transient positivity. ANA was positive in 8/34 (23.5%), mostly low-titer. aPL was tested in only 43% of the cohort, with higher positivity in the prolonged-fever group (10/10 vs. 4/7), suggesting selection bias and likely overestimating true prevalence (15). Notably, transient autoantibodies during acute Q fever do not indicate antiphospholipid syndrome or SLE. Rapid 12-week negativity supports an infection-induced, self-limited phenomenon. Thus, positive autoantibodies in acute febrile illness should not prompt autoimmune workup; a 12-week follow-up can distinguish transient from persistent positivity. The strong association between aPL positivity and prolonged fever (100% vs. 57%) may mark immune hyperactivation rather than direct pathogenicity, linking transient autoimmunity to clinical outcomes.

Risk factors for prolonged fever. Median fever duration after doxycycline was 8 days (range 1–33). Using a 7-day cutoff, 22 patients (53.7%) had prolonged fever (>7 days). Compared to the non-prolonged group (≤7 days, n=19), the prolonged group had significantly higher WBC, CRP, and ESR, as well as lower CD8+ T and B cell counts. aPL positivity was also higher (10/10 vs. 4/7, p=0.023). These associations suggest a phenotype of intense inflammation and relative lymphopenia. particularly of CD8+ T cells which are critical for controlling C. burnetii (20). Collectively, these findings indicate that immune dysregulation-rather than pathogen load (similar mNGS reads in both groups)-drives delayed recovery. However, due to the cross-sectional design, we cannot determine whether lymphopenia precedes or results from sustained inflammation (e.g., via cytokine-mediated suppression or apoptosis). No differences were observed in age, sex, platelet count, D-dimer, or mNGS read counts, indicating that pathogen load alone does not predict fever duration.

Organ involvement. The lungs were the most commonly involved organ (51.2%), consistent with aerosol transmission and tropism for alveolar macrophages (14, 21). Pleural and peritoneal effusions, observed in a substantial minority, reflect hematogenous dissemination and serosal inflammation. Among five patients undergoing lumbar puncture for severe headache, three had elevated CSF opening pressure, but significant pleocytosis or biochemical abnormalities were absent, suggesting headache may result from systemic inflammation or cytokine release rather than classic meningitis (22).

### Clinical value of plasma mNGS

In this study, plasma mNGS enabled specific diagnosis of C. burnetii infection in patients with undifferentiated febrile illness, many of whom had negative or inconclusive routine test results. Unlike serology (which requires convalescent sampling) or pathogen-specific PCR (which relies on clinical suspicion), mNGS offers an unbiased, rapid detection method for rare or unexpected pathogens. Although a direct comparison with serology/PCR was not possible due to their unavailability in this retrospective cohort, our experience supports the practical utility of plasma mNGS for early diagnosis of acute Q fever in settings where conventional methods are limited or negative.

This study has several limitations. (1) Selection bias: The retrospective, single-center design included only patients who underwent plasma mNGS testing. At our center, mNGS was ordered for patients with inconclusive routine tests, atypical presentations, or failed empirical therapy; thus, our cohort likely represents a more severe or diagnostically challenging subgroup, and the findings should be interpreted accordingly. (2) Lack of independent confirmatory testing (e.g., C. burnetii-specific PCR or serology); therefore, case confirmation relied on a composite of plasma mNGS, clinical manifestations, and epidemiological exposure history, which may have introduced misclassification bias. (3) Small sample size, particularly for subgroup analyses, limits statistical power. (4) Incomplete laboratory parameters due to retrospective data collection. (5) No serial measurements of lymphocyte subsets, autoantibodies, or cytokines (e.g., IL-6, IL-10, TGF-β) were performed, precluding temporal and mechanistic insights. (6) No validation cohort to confirm the identified risk factors. These limitations are inherent to the retrospective design and have been acknowledged throughout the manuscript. Prospective studies with serial cytokine profiling and immune monitoring are needed to clarify the causal relationships between cytokine dynamics, lymphopenia, and fever resolution in acute Q fever.

## Conclusion

Acute Q fever presents diverse manifestations and is frequently associated with transient autoantibody positivity (e.g., aPL, ANA). Prolonged fever after targeted antibiotic therapy is associated with elevated acute−phase reactants, reduced CD8+ and B lymphocytes, and aPL positivity. These findings suggest an association between immune dysregulation/transient autoimmunity and fever duration, but causal inferences cannot be drawn given the observational and retrospective nature of the study.

## Data Availability

The raw sequencing data for 34 of the 44 patients included in this study have been deposited in the NCBI Sequence Read Archive (SRA) under BioProject PRJNA1470068. Data for the remaining 10 patients are unavailable due to loss of original records by the manufacturer. We have verified that the 34 available cases fully support all reported conclusions, and the key findings remain robust. The datasets analyzed during the current study are available from the corresponding author on reasonable request.
